# Nonsynaptic junctions on myelinating glia promote preferential myelination of electrically active axons

**DOI:** 10.1038/ncomms8844

**Published:** 2015-08-04

**Authors:** Hiroaki Wake, Fernando C. Ortiz, Dong Ho Woo, Philip R. Lee, María Cecilia Angulo, R. Douglas Fields

**Affiliations:** 1 Section on Nervous System Development and Plasticity, National Institutes of Health, National Institute of Child Health and Human Development, Building 35, Room 2A211, MSC 3713, 35 Lincoln Drive, Bethesda, Maryland 20892, USA; 2 INSERM U1128, 75006 Paris, France; 3 Université Paris Descartes, Sorbonne Paris Cité, 75006 Paris, France

## Abstract

The myelin sheath on vertebrate axons is critical for neural impulse transmission, but whether electrically active axons are preferentially myelinated by glial cells, and if so, whether axo-glial synapses are involved, are long-standing questions of significance to nervous system development, plasticity and disease. Here we show using an *in vitro* system that oligodendrocytes preferentially myelinate electrically active axons, but synapses from axons onto myelin-forming oligodendroglial cells are not required. Instead, vesicular release at nonsynaptic axo-glial junctions induces myelination. Axons releasing neurotransmitter from vesicles that accumulate in axon varicosities induces a local rise in cytoplasmic calcium in glial cell processes at these nonsynaptic functional junctions, and this signalling stimulates local translation of myelin basic protein to initiate myelination.

## Abstract (web summary)

The myelin sheath on vertebrate axons is critical for neural impulse transmission, but whether electrically active axons are preferentially myelinated by glial cells, is not clear. Here the authors show that cultured oligodendrocytes preferentially myelinate electrically active axons via a mechanism dependent on nonsynaptic vesicular release of glutamate.

## Graphical abstract

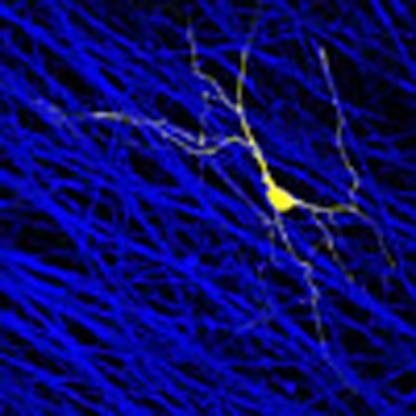

The surprising discovery of synapses formed on glial progenitors, oligodendrocyte progenitor cells (OPCs, also called NG2 cells) has remained enigmatic for over a decade^1^. These cells mature to form myelin insulation on axons^2^,^3^, and several functions for synapses on OPCs have been proposed^4^. A leading hypothesis is that axon-OPC synapses may stimulate myelination selectively on electrically active axons to increase the speed of impulse transmission through electrically active neural circuits^5^,^6^. This would have significant effects on neural circuit function. Since myelination continues in many brain regions through early life, preferential myelination of electrically active axons could enable environmental factors to modify neural circuit development according to functional experience^7^.

Synapses on OPCs could increase myelination in an activity-dependent manner in several ways, including promoting OPCs to differentiate into mature oligodendrocytes or by increasing OPC survival or proliferation. However, signals from axons must also regulate initiation of myelin wrapping even after OPCs have matured, because mature oligodendrocytes can be associated with axons early in development but not form myelin until much later in prenatal or adult life^8^. It has been shown that vesicular release of glutamate from axons stimulates local translation of myelin basic protein (MBP) and stimulates myelin induction^9^. This signalling could be mediated by synaptic transmission or by spillover of neurotransmitter from axo-glial synapses activating extrasynaptic glutamate receptors on OPC processes^10^,^11^.

Alternatively to synaptic transmission, other forms of axo-glial communication could signal electrical activity in axons to OPCs. Nonsynaptic release of neurotransmitter operates by both vesicular and non-vesicular release mechanisms. Neurotransmitters can be released in the absence of morphological synaptic contacts to activate neurotransmitter receptors on other cells (volume conduction)^12^. In contrast to synaptic communication, which is a specialization for rapid (millisecond) and highly point-to-point localized signalling between axons and dendrites, volume transmission could be particularly well suited for communication between axons and myelinating glia^13^. Vesicle fusion is seen at axonal swellings (varicosities) that lack identifying features of a synapse. Notably missing are the close apposition of pre- and post-synaptic membranes, submembrane thickening caused by cytoskeletal proteins that organize neurotransmitter receptors and intracellular signalling molecules in the postsynaptic apparatus and the focused accumulation of synaptic vesicles docked at the presynaptic membrane. Neurotransmitter signalling at axonal varicosities along nerve fibres is characteristic of autonomic transmission in the enteric nervous system^14^ and cholinergic transmission in neocortex^15^, but most neurons have similar axon varicosities. In addition to nonsynaptic vesicular release, neurotransmitters also can be released along axons through membrane channels^16^.

Another important question is if given a choice, will oligodendroglial cells preferentially myelinate electrically active axons? In addition, oligodendrocytes are multipolar cells but it is unknown how different branches of the same oligodencrocyte are instructed by axons to act autonomously and selectively synthesize myelin in those processes that are in contact with active axons. In the present experiments, calcium imaging, electron microscopy and electrophysiology were used to determine the involvement of axon-glia communication in myelination of electrically active axons *in vitro*. The results indicate a strong preference for oligodendrocytes to myelinate electrically active axons via a mechanism dependent on nonsynaptic vesicular release of glutamate but independent of synapses on OPCs.

## Results

### Preferential myelination of electrically active axons

**Figure 1:**
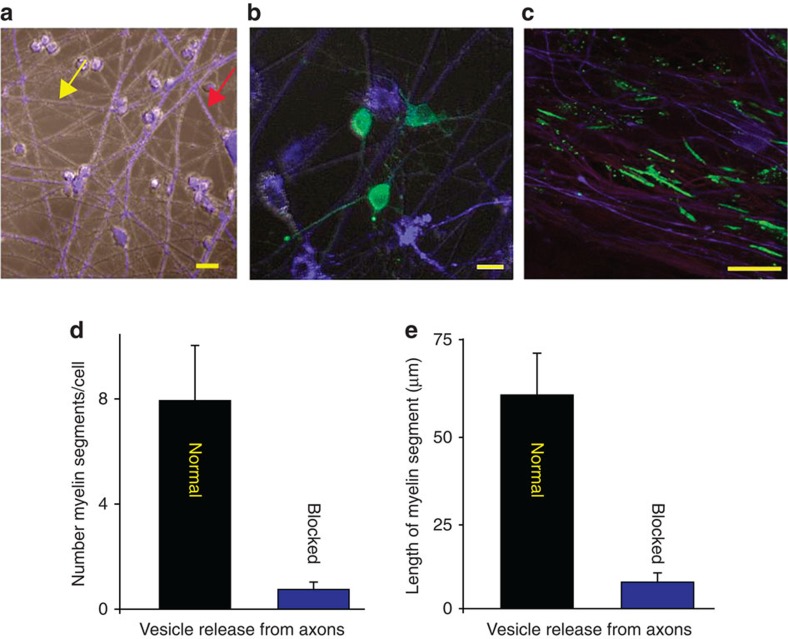
Electrically active axons releasing synaptic vesicles are preferentially myelinated. (**a**) DRG neurons treated with BoNT/A and stained with cell tracker (blue, see red arrow) co-cultured with normal (untreated) neurons (grey, see yellow arrow). (**b**) OPCs (green, GCaMP3) were added to the cultures to determine whether exocytosis of neurotransmitter from axons influenced myelination. (**c**) Axons were stimulated for 9 s at 10 Hz every 5 min for 10 h and cultured for 3 weeks. Myelin (green, myelin basic protein, MBP) analysed 3 weeks after co-culture formed preferentially on axons releasing synaptic vesicles (purple, neurofilament), and (**d**) number of myelin segments/cell were more in normal axons (*P*<0.001, *n*=7 cells from four dishes) (**e**) myelin segments were also longer in normal axons (*P*<0.001, *n*=9 cells from four dishes). Scale bar, 10 μm (**a**,**b**); 20 μm (**c**).

The hypothesis that axons that are electrically active would be preferentially myelinated was tested by co-culturing OPCs with neurons that could release synaptic vesicles together with other neurons in which vesicular release was blocked. Dorsal root ganglion (DRG) neurons were used in these studies because they have several advantages. DRG neurons have no dendrites and thus they are ideal for studying oligodendrocyte interactions with axons. The long central axons of DRG neurons are myelinated by oligodendrocytes, and DRG neurons do not form synapses on themselves (*in vivo* or in culture)^17^,^18^. DRG neurons do not fire action potentials spontaneously, and they fire a single action potential in response to a brief electrical stimulus; thus, the firing frequency and pattern can be regulated precisely by electrical stimulation of neurons in cell culture. In these experiments, half of the neurons were treated with the clostridial neurotoxin, botulinum A (BoNT/A) together with a blue dye to identify these axons. BoNT/A is a potent and highly selective enzyme that cleaves synaptosome-associated protein-25 (SNAP-25), the t-SNARE (Target membrane-associated soluble N-ethylmaleimide-sensitive factor attachment protein receptor) necessary for neurotransmitter release from synaptic vesicles. The other half of the neurons were untreated, providing OPCs a choice as to which axons to myelinate once undergoing differentiation. After washing out the toxin, which continues to inhibit neurotransmitter release for at least 4 weeks^19^, OPCs were added to neuronal cultures containing normal and BoNT/A-treated neurons to determine whether myelin formed preferentially on axons that release synaptic vesicles in response to electrical stimulation (Fig. 1a,b; Supplementary Fig. 1b–d). Compact myelin was identified by immunocytochemistry for MBP 3 weeks after culturing OPCs on DRG axons.

Consistent with the hypothesis, the results showed that when given a choice, oligodendrocytes preferentially myelinated axons that could release synaptic vesicles (Fig. 1c). By far, the majority of myelin segments (10 times more) were found on control axons compared with axons in the same culture in which vesicular release was inhibited (Fig. 1d) (*P*<0.001, *t*-test *n*=7 cells from four dishes). When myelin did form on axons that were unable to release synaptic vesicles, individual myelin segments were only 1/8 as long as normal (Fig. 1e, *P*<0.001, *t*-test, *n*=9 cells from four dishes). Thus, oligodendroglia preferentially myelinate electrically active axons in these experiments by a mechanism dependent on exocytosis. This is consistent with the hypothesis that synaptic transmission between axons and OPCs promotes the initial events of myelination. However, release of neurotransmitter along axons can also take place in the absence of synapses, providing an alternative explanation for this result.

### Axo-glial synaptic transmission in myelination

**Figure 2:**
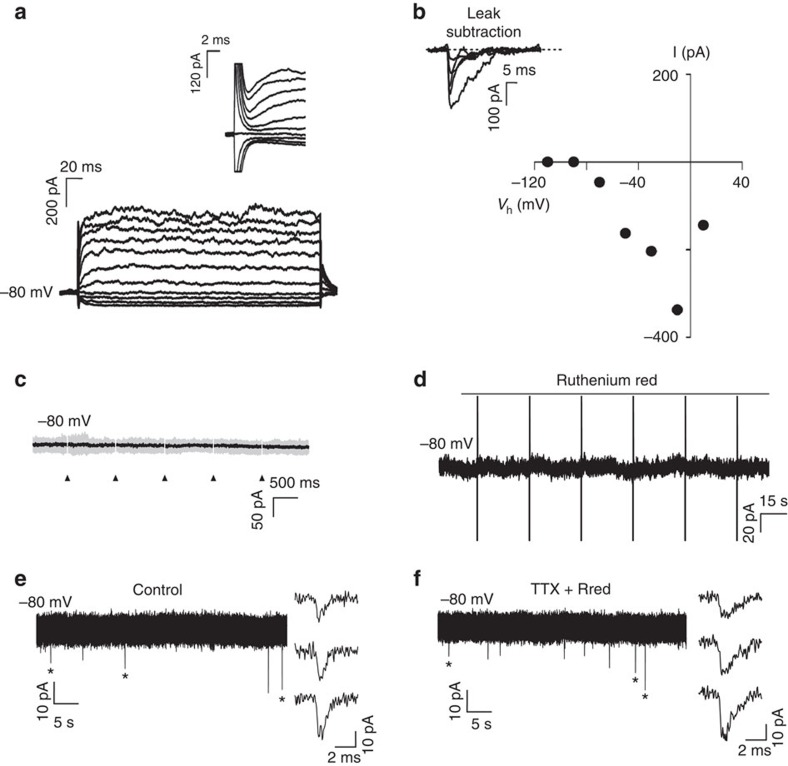
Myelination occurs in absence of axon-OPC AMPAR-mediated synaptic currents. (**a**) Currents elicited in a recorded OPC held at −80 mV 1 day after plating by voltage steps from +20 to −110 mV. Note the presence of sodium currents (inset). (**b**) *I*–*V* curve of sodium currents for the same OPC after leak subtraction (inset). (**c**) Lack of evoked synaptic currents in an OPC upon DRG axonal stimulation. There was no response in 74 cells tested (unstimulated *n*=17 cells, from seven dishes; pre-stimulated electrically with (*n*=15 cells from six dishes) or without (*n*=42 from 17 dishes) BoNT/A). Individual (grey, 15 sweeps) and average traces (black) are shown. Axonal stimulation time is indicated with arrowheads. (**d**) Bath application of the secretagogue ruthenium red (75 μM) in absence of electrical DRG axonal stimulation did not evoke any currents in the same OPC. Capacitive currents in response to a test pulse are shown (unstimulated *n*=10 cells from four dishes, pre-stimulated *n*=18 cells from seven dishes and pre-stimulated with BoNT/A *n*=7 cells from four dishes). (**e**,**f**) Spontaneous (**e**) and miniature (**f**) synaptic currents in an OPC recorded at 15 postnatal days in acute coronal corpus callosum slices of NG2-DsRed mice (*N*=2 mice). Miniature synaptic currents were recorded in 1 μM tetrodotoxin (TTX) and 75 μM ruthenium red (Rred). The mean frequency, rise and decay times of spontaneous synaptic activity are 0.51 Hz, *t*_10–90%_=332 μs and *τ*=1.35 ms, respectively (insets, *n*=7 cells from seven different brain slices). The holding potential is indicated for each trace.

Preferential myelination of electrically active axons could result from synaptic communication stimulating OPC development; however, further experimental results did not support this. Supplementary Fig. 1a shows typical OPC morphology in co-culture for 24 h with DRG neurons. In our conditions, monocultured OPCs did not exhibit inward sodium currents until 2–3 days *in vitro* (d.i.v.) (0 of 8 cells at 0–1 d.i.v. 5 of 10 at 2–3 d.i.v.; Supplementary Fig. 2b,c). Interestingly, we observed that contact with axons greatly accelerated the onset of inward sodium current expression in OPCs. Sodium currents were evident in 84% of 91 recorded cells from the first day of co-culture with DRG neurons, independently of the treatment (Fig. 2a,b; Supplementary Fig. 2e). No changes on *I*–*V* curves for sodium or potassium currents, input resistance or capacitance of OPCs were observed in co-cultures unstimulated and pre-stimulated electrically with or without BoNT/A (Supplementary Fig. 2d–g).

To test for the presence of synaptic currents in OPCs co-cultured with neurons, electrical stimulation (1 Hz; Supplementary Fig. 2a) was applied through extracellular electrodes to depolarize DRG axons that traverse beneath a high-resistance barrier separating DRG cell bodies and axons in different compartments of three-compartment chambers (Campenot chambers). To test for the presence of evoked synaptic currents in OPCs cultured on axons in the central compartment, we recorded these cells in whole-cell configuration at a holding potential of −80 mV while stimulating DRG neurons through the extracellular electrodes. Importantly, evoked AMPAR (α-amino-3-hydroxy-5-methyl-4-isoxazole propionic acid receptor)-mediated currents in OPCs were never detected in co-cultures, regardless of whether OPCs were in contact with axons that were previously unstimulated (*n*=17), or axons that were pre-stimulated electrically with (*n*=15) or without (*n*=42) BoNT/A (Fig. 2c). To corroborate the lack of axon-OPC synapses, we analysed the possible existence of spontaneous synaptic currents in OPCs recorded before and during application of the secretagogue ruthenium red, which stimulates a massive release of synaptic vesicles. No spontaneous synaptic currents were detected in OPCs either in control conditions or in response to application of ruthenium red (unstimulated *n*=10; pre-stimulated *n*=18; pre-stimulated with BoNT/A *n*=7) (Fig. 2d). It is noteworthy that most OPCs (80%) recorded during extracellular stimulation and/or in ruthenium red had a membrane resistance >300 MΩ, sufficient for the detection of small or distant synaptic currents in these progenitors^11^ (Supplementary Fig. 2f). In addition, confirming previous research, we find that AMPAR-mediated synaptic currents are common in recordings of OPCs in acute brain slices, using the same recording conditions that were used in co-cultures (Fig. 2e,f). Since myelination is promoted by electrical stimulation in co-cultures^20^,^21^, our results indicate that activity-dependent regulation of myelination does not require AMPAR-mediated synaptic communication between axons and OPCs. Therefore the hypothesis that synapses are required for formation of myelin on electrically active axons is not supported.

### Nonsynaptic communication in activity-dependent myelination

Next, we wished to determine how OPCs could selectively myelinate axons that were electrically active in the absence of synapses from axons. DRG neurons do not form synapses in monoculture^17^,^18^; however, vesicle recycling in monoculture DRG neurons occurs along axons in response to electrical stimulation as shown by monitoring the fluorescent indicator FM 4–64 (Supplementary Fig. 1e). Since treatment with botulinum toxin strongly inhibited both vesicle recycling along axons induced by electrical stimulation^9^ and myelination independent of synaptic transmission (Figs 1 and 2), we hypothesized that nonsynaptic vesicular release of neurotransmitter along axons may stimulate myelination.

**Figure 3:**
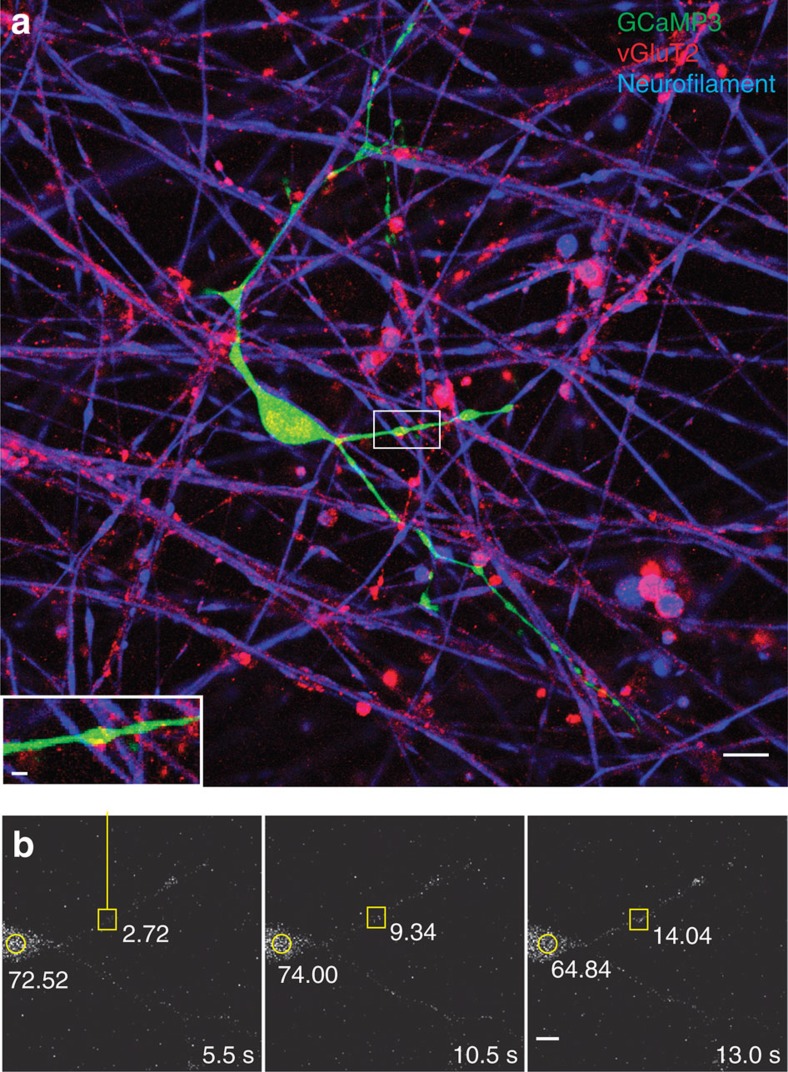
Ca^2+^ increases in processes of OPCs at axonal varicosities. (**a**) OPC processes (green, GCaMP3 transfection) form specialized functional junctions with DRG axons (blue, neurofilament), containing accumulations of synaptic vesicles containing glutamate (red, vGluT2). Scale bar, 10 μm. After live-cell calcium imaging (**b**), the cultures were fixed and stained by immunocytochemistry to determine whether that the calcium responses were associated with axo-glial contacts containing glutamatergic synaptic vesicles (inset in **a**). Note colocalization between axonal varicosity (red, vGluT2) and swellings in OPC process (green, GCaMP3). Scale bar, 2 μm. (**b**) Time-lapse series showing a local increase in Ca^2+^ in OPC-axon junctions in response to electrical stimulation of axons. A stimulus-induced, local increase in Ca^2+^ in the same axo-glial junction (yellow square) that is shown in **a** is recorded in the OPC transfected with GCaMP3. Time since stimulus onset is shown in each frame (5.5–13 s). The fluorescence intensities at the axo-glial contact (yellow square) and the OPC cell body (yellow circle) are shown. Note the local increase in fluorescence intensity at the axo-glial junction after stimulation but this is not accompanied by an increase in fluorescence intensity in the soma. Scale bar, 5 μm.

To test this possibility, we imaged cytoplasmic calcium responses in OPCs transfected with the genetic calcium indicator GCaMP3 in response to electrical stimulation of axons. The results showed functional communication between axons and OPCs despite the absence of synaptic currents (Fig. 3). Ca^2+^ transients induced in OPCs by axonal action potentials were typically seen at points where OPC processes were associated with axons (Fig. 3, insets).

**Figure 4:**
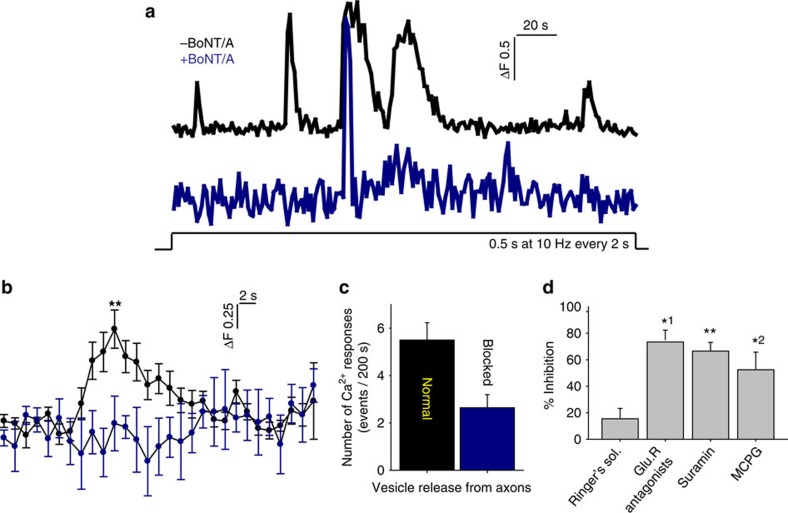
Ca^2+^ increase in OPC processes is mediated by glutamate and ATP released in response to action potentials in axons. (**a**) Representative Ca^2+^ traces in OPC processes in response to electrical stimulation of axons without (black) and with (blue) BoNT/A treatment to block SNARE-dependent exocytosis. (**b**) Averaged Ca^2+^ traces in OPCs from 14 dishes are shown. Responses were inhibited by BoNT/A treatment. **P=0.001, *t*-test, peak amplitude, *n*=14 cells from 14 dishes with no BoNT/A, *n*=14 cells from 14 dishes with BoNT/A. (**c**) Number of Ca^2+^ responses were reduced significantly by BoNT/A treatment (blocked). The peak Ca^2+^ response within 200 s of axonal stimulation (10 Hz) were measured. *P*=0.004, *t*-test, *n*=14 cells from 14 dishes with no BoNT/A, *n*=14 cells from 14 dishes with BoNT/A. (**d**) Summary graph shows per cent inhibition of Ca^2+^ responses following treatment with selective blockers of glutamatergic and purinergic neurotransmitter receptors, including a combination of CNQX, DAPV, MCPG (GluR antagonists), suramin and MCPG. The results implicate both glutamate and ATP neurotransmitter signalling. *P*=0.3, *n*=6 cells from six dishes, *^1^*P*=0.03, *n*=4 cells from four dishes, **P=0.008, *n*=5 cells from five dishes, *^2^*P*=0.02, *n*=6 cells from six dishes, all paired *t*-test.

The onset latency of electrically induced calcium rise in OPCs was much longer than synaptic transmission; >1 s. In response to pulsed stimulation (0.5 s at 10 Hz every 2 s), the average time to peak of the first response was 25±5.7 s (*n*=14 dishes), with multiple subsequent calcium responses during stimulation sustained for 200 s (Fig. 4a,b). Application of BoNT/A significantly reduced the amplitude of electrically induced calcium response in OPCs (Fig. 4a,b). Selective inhibitors of neurotransmitter receptors indicate that these responses were mediated by both glutamatergic and purinergic signalling. A cocktail of glutamate receptor antagonists inhibiting NMDA (*N*-methyl-D-aspartate), mGluR and AMPA glutamate receptors (DAPV (D-(−)-2-amino-5-phosphonopentanoic acid), MCPG ((RS)-α-methyl-4-carboxyphenylglycine) and CNQX (6-cyano-7-nitroquinoxaline-2,3-dione), respectively) reduced the amplitude of electrically evoked Ca^2+^ in OPCs by 80% (Fig. 4d). Suramin inhibition of purinergic receptors, or MCPG inhibition of mGluR receptors alone, reduced the amplitude of responses significantly (Fig. 4d). These results implicate vesicular release of ATP and glutamate from varicosities in signalling to OPC processes.

**Figure 5:**
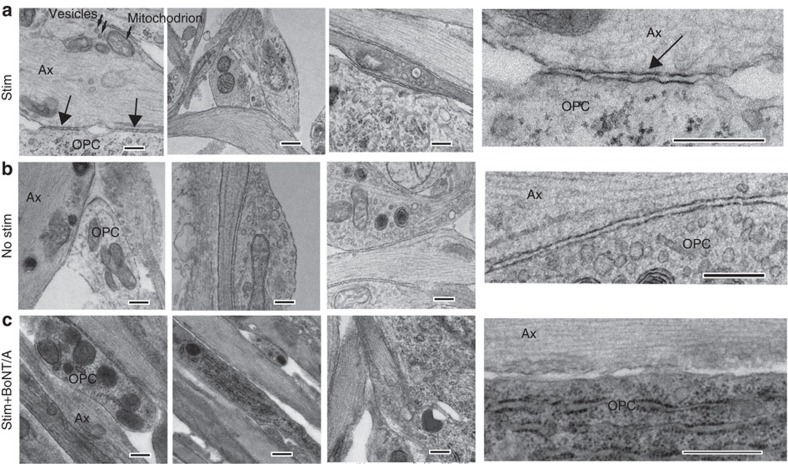
Nonsynaptic junctions between axons and OPCs are promoted by vesicular release from axons. Transmission electron microscopy showed specialized contacts (arrows) between OPC processes (OPC) and axon varicosities (Ax) containing intracellular vesicles (small arrows in **a**) and mitochondrion, but no synapses were detected. Three examples for each condition are shown. Insets (right column) show these junctions at higher magnification. Such contacts were evident in cultures stimulated for 9 s at 10 Hz every 5 min for 10 h (Stim) before plating OPCs, and unstimulated cultures (**b**), but such contacts were not found in stimulated cultures treated with BoNT/A before adding OPCs (**c**). Scale bar, 1 μm.

**Figure 6:**
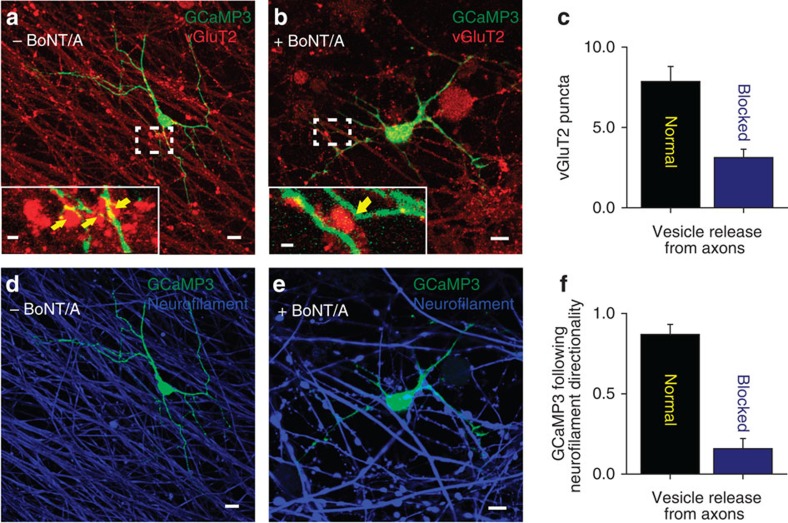
OPC processes preferentially contact electrically active axons releasing synaptic vesicles and form nonsynaptic axo-glial junctions. (**a**,**b**) Images showing OPCs transfected with GCaMP3 construct (green) and vGluT2 immunocytochemistry (red) to identify glutamate-containing vesicles in axons. OPCs were plated on axons either previously treated (**b**) or not treated (**a**) with BoNT/A. (**a**,**b**) Magnified views of glutamate-containing vesicles and OPC processes. Yellow arrows indicate vGluT2 stained puncta in close opposition to GCaMP3 processes. (**c**) The number of vGluT2 puncta >0.5 μm in diameter was reduced significantly on axons previously treated with BoNT/A (*P*=0.0005, *t*-test, *n*=7 cells from seven dishes with no BoNT/A, *n*=8 cells from eight dishes with BoNT/A). (**d**,**e**) Images showing OPC transfected with GCaMP3 construct (green) and immunocytochemistry for neurofilament (blue, an axon marker). (**f**) Summary graph showing that the fraction of all OPC processes (**d**,**e**) in individual OPCs forming parallel associations with axons was significantly reduced when axons were previously treated with BoNT/A (*P*=0.0002, *t*-test, *n*=7 cells from seven dishes with no BoNT/A, *n*=8 cells from eight dishes with BoNT/A). Scale bars, 10 μm (**a**, **b**,**d**,**e**); 2 μm (**a**,**b**).

Transmission electron microscopy showed sites of intercellular membrane specialization at sites of contact between axons and OPCs (Fig. 5a), but these were not found in OPCs on axons in which vesicular release had been blocked (Fig. 5c). This suggests that exocytosis from axons promotes the formation of specialized junctions between axons and OPC processes. Such junctions could also be found in the absence of BoNT/A—without electrical stimulation (Fig. 5b), suggesting spontaneous release of vesicles allows formation of the axo-glial contacts. These contacts lacked the characteristic features of synapses^1^,^22^, including a postsynaptic density, uniform intercellular space between 20 and 30 nm (synaptic cleft) and accumulation of synaptic vesicles docked to the electron dense active zone. Rather, the OPC contacts were associated with axon varicosities, swellings along unmyelinated axons that contain vesicles of various sizes and mitochondria. Immunocytochemical localization of the vesicular glutamate transporter 2 (vGluT2) was used to identify glutamate-containing vesicles in axons, which were found to accumulate in axon varicosities. The number of vGluT2 puncta in axons that co-localized along OPC processes (visualized by transfection with GCaMP3) was significantly lower on axons that were treated with BoNT/A (Fig. 6a–c; *P*=0.005, *t*-test, *n*=15 cells from 15 dishes). This indicates that exocytosis from axons promotes the formation of the glutamatergic axo-glial junctions at axon varicosities.

Interestingly, OPC processes were less closely associated with axons in which exocytosis was inhibited by BoNT/A treatment. Rather than adhering to the axon and tracking along it, OPC processes frequently intersected and crossed over axons rather than running parallel together with the axon (Fig. 6d–f; *P*=0.0002, *t*-test, *n*=15 cells from 15 dishes).

Formation of specialized junctions between axons and oligodendrocytes, which were described as ‘spot welds' in the earliest electron microscopic study of central nervous system myelination, is the first event in myelin formation^23^. Importantly, these junctions shown here in cell culture and previously *in vivo*^23^,^24^ lack ultrastructural specializations characteristic of synaptic junctions. In line with these anatomical observations, our findings show that specialized nonsynaptic axon-OPC junctions are functional and signal via vesicular release from axon varicosities that induces Ca^2+^ increases in OPCs and may serve to promote myelination.

### Autonomous oligodendrocyte processes in activity-dependent myelination

**Figure 7:**
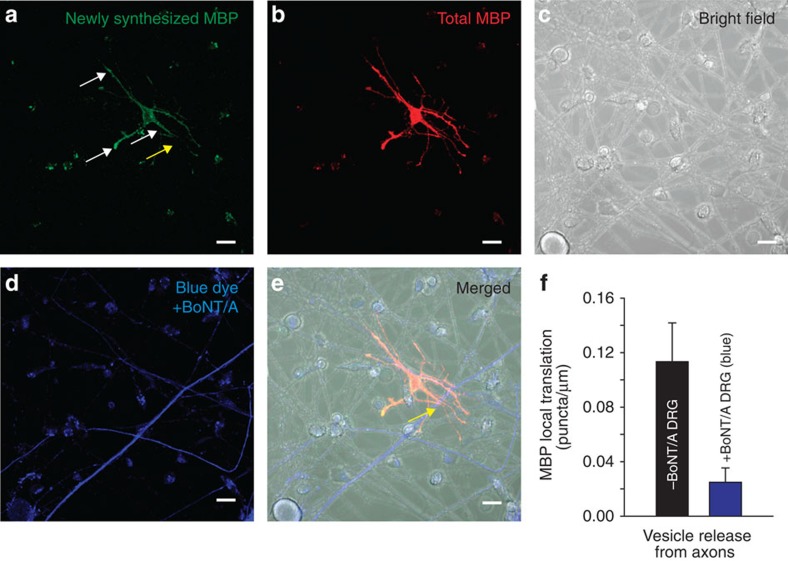
Local translation of MBP occurs preferentially on OPC processes contacting electrically active axons releasing synaptic vesicles. (**a**) Newly synthesized MBP (green). Axons were stimulated electrically at 10 Hz for 10 min and local translation of MBP was monitored using kikume MBP fluorescence after 40 min. White arrows indicate new MBP translation in contrast yellow arrow showing no new MBP translation. (**b**) Total MBP (red). (**c**) Bright field showing cell morphology of co-culture. (**d**) Axons treated previously with BoNT/A to block synaptic vesicle release (blue). (**e**) Combined images from **a**–**d**. Differential interference contrast and fluorescence to identify axons treated (blue, yellow arrow) and not treated with BoNT/A. Yellow arrow shows axon and OPC interaction without MBP translation (notice yellow arrow in **a** with lack of new MBP green puncta indicating no new MBP translation). (**f**) Quantification shows significantly more local translation of MBP in OPC processes that were in contact with axons releasing synaptic vesicles (grey axons) (0.11±0.024 versus 0.028±0.011 puncta per micrometre length of axon not treated or previously treated with BoNT/A respectively, *P*<0.001, *t*-test, *n*=24 cells from non-blue, *n*=23 cells from blue axons, five dishes). Scale bar, 10 μm.

Oligodendrocytes are multipolar cells that can myelinate up to 50 different axons. Axonal factors are known to induce myelination, but it is unclear whether individual cell processes of an oligodendrocyte can act autonomously to myelinate different axons, or whether oligodendrocyte commitment to myelin induction is a cell-wide event. Local translation of myelin basic protein is stimulated in OPCs by glutamate released from axons acting on mGluR and NMDA receptors on oligodendrocytes^9^ but it is unknown whether this occurs selectively in those processes of an OPC that are in contact with electrically active axons. To answer this question, OPCs were transfected with kikume MBP-3′-untranslated region, a photo-activated fluorophore that enables identification of newly synthesized protein by a change in fluorescence from red to green after photoactivation. Local translation of MBP was found to be preferentially induced in OPC processes in contact with electrically active axons compared with OPC processes from the same cell in contact with axons in which vesicular release had been inhibited by prior treatment with BoNT/A (Fig. 7). Thus, different cell processes of an oligodendrocyte act independently in myelin induction and thus are able to compartmentalize signals. We conclude that one of the axonal factors determining whether different branches of an oligodendrocyte myelinate an axon is the activity-dependent exocytosis of vesicles containing neurotransmitter along the fibre.

## Discussion

The experiments provide several novel findings: When provided a choice, OPCs preferentially myelinate electrically active axons releasing synaptic vesicles. This activity-dependent communication between axons and OPCs is mediated by nonsynaptic intercellular junctions that signal through intracellular calcium. Myelin forms normally and is stimulated by electrical activity in axons in culture even though there is no evidence of synapses by electrophysiological and electron microscopic analysis. Individual cell processes of oligodendrocytes act autonomously, initiating myelination in response to electrical activity in axons in contact with the oligodendroglial process, which stimulates local translation of MBP in that subcellular domain.

In conclusion, myelin is formed preferentially on electrically active axons releasing vesicles, but synapses on OPCs are not required for activity-dependent stimulation of myelination. Local calcium signalling produced by vesicular release of glutamate is well suited to local subcellular control of myelin induction in individual OPC processes, by activating glutamate receptors localized in the fine processes closely associated with axons^9^. ATP release can also occur from synapses and mediate discrete signals, but purinergic signalling appears to be more relevant to modulating differentiation and proliferation of OPCs, in part because these signalling pathways activate global intracellular calcium responses in the cell^9^. This is possibly associated with wider spread release of ATP from axons through volume-regulated anion channels^16^, and differences in localization of glutamate and purinergic receptors in the cell.

Preferential myelin induction on electrically active axons would have profound effects on circuit function by the resulting increased conduction velocity, and thus provide another mechanism of plasticity complementing synaptic plasticity^7^. In human brain imaging studies, white matter structure is affected by learning^25^, and recent studies show that social isolation impairs myelin formation in the forebrain of mice^26^,^27^; the present findings could provide a cellular mechanism participating in the effects of environmental experience on myelination. These new findings may also have implications for disease, including psychiatric illness and impaired remyelination after conduction block in multiple sclerosis.

## Methods

### Cell culture

All experiments were conducted in accordance with animal study protocols approved by the NICHD Animal Care and Use Committee. DRG neurons were dissected from the spinal cords of embryonic day-13.5 mice as described^9^. Neurons were grown for ∼4 weeks in MEM media supplemented with N3 containing 100 ng ml^−1^ of nerve growth factor and 5% heat-inactivated horse serum in the side compartments of three-compartment chambers equipped with stimulating electrodes^28^ or on coverslips that were coated with poly-L-lysine and collagen. Mitosis of non-neuronal cells was inhibited by a 4-day treatment with 13 μg ml^−1^ fluoro-2′-deoxyuridine beginning 1 day after plating. These cultures can be maintained indefinitely with half-volume changes of media every 3 days. Primary cultures of OPCs were obtained from cerebral cortices of embryonic day-19 P2 rats and plated into 75-cm^2^ tissue culture flasks. The resulting cultures were maintained at 5% CO_2_ and 37 °C in media containing 10% fetal bovine serum (Life Technologies, Carlsbad, CA, USA). After 11 days in culture, the flasks were shaken at 37 °C for 1 h to kill non-glial cells and remove microglia, then the media was changed and the flasks were shaken overnight to lift OPCs from the flask. To enrich for OPCs, the cell suspension was pelleted, resuspended and incubated in an uncoated culture dish for 30 min. Contaminating cells, primarily endothelial cells, astrocytes, macrophages and microglia adhere strongly to the plastic and can be separated out by this panning method. For myelinated co-cultures, purified OPCs (>90% OPCs) were counted and plated on 3–4-week-old DRG cultures at a density of 40,000 cells per side compartment. After 3–4 h, the media was removed and replaced with N1 differentiating media. Action potentials were induced in DRG axons by a 200-μs 5-V biphasic pulse through platinum electrodes in three-compartment chambers^28^. Axons growing into the central compartment beneath the high-resistant barriers are stimulated but cells in the lateral compartments are not depolarized. For 3-week co-culturing myelination experiments, stimulation was applied in 0.5 s bursts at 10 Hz every 2 and 15 s bursts at 10 Hz every 5 min, using biphasic pulses (200-μs 6 V, square-wave pulse) for 5 h (ref. 9). For Ca^2+^ imaging, field stimulation (15 s at 10 Hz, 30–50 V) was delivered through two 1-cm-long horizontal platinum electrodes in cultures of DRG neurons grown on 25-mm coverslips.

### Intracellular Ca^2+^ imaging

OPCs transfected with GCaMP3 were imaged during electrical stimulation using a confocal microscope equipped with a 40 × (1.3 numerical aperture, NA) objective lens, excitation at 488 nm by scanning laser, and emission light filtered through a HQ528/50 splitter/filter. Quantification of images was performed using Image J software (NIH).

### Vesicle recycling

Recycling synaptic vesicles in DRG axons were labelled with FM 4–64 (Life Technologies). To stain the total pool of recycling vesicles, axons were loaded by electrical stimulation (10 Hz, 30 V, 5 ms, biphasic square wave) for 20 s in media containing 2.5 μM FM 4–64 dye. The dye was allowed to remain on the cells for 60 s after cessation of the stimulus to permit complete compensatory endocytosis, and was subsequently removed during a 7-min period with eight complete solution changes.

### Pharmacology

Drugs were added directly to the culture dish before experiments. Botulinum toxin A, kindly provided by E. A. Johnson, was added to cultures at a final concentration of 3 nM for at least 18 h before experiments to block vesicular release^19^. Immunoblotting confirmed cleavage of SNAP-25 in DRG neurons treated with BoNT/A. Previous studies show that block of neurosecretion from DRG neurons in cell culture occurs within 4 h of BoNT/A and last at least 4 weeks^19^. MCPG (500 μM), DAPV (50 μM), CNQX (20 μM), and suramin (50 μM) were obtained from Tocris (Ellisville, MO, USA). Cell Tracker Blue CMAC Dye is from Life Technologies (NY, USA).

### Confocal microscopy

All images were acquired on a Zeiss 510 NLO confocal microscope (Carl Zeiss MicroImaging, Inc. Thornwood, NY, USA) equipped with both 40 × (1.3 NA) and 63 × (1.4 NA) oil-immersion lenses using appropriate laser lines and excitation/emission filters. For live-cell imaging, coverslips were mounted in an imaging chamber and continuously superfused with sterile-filtered saline. Photoactivation of Kikume was performed with ultraviolet lamp exposure for >10 min.

### Antibodies

The antibodies used were as follows: vGluT2 (1:1,000, Cat. AB2251, Millipore, Billerica, MA, USA), neurofilament (1:5,000, Cat. NFH, Aves Labs, Tigard, Oregon), MBP (1:1,000, Cat. SMI-99, Sternberger Monoclonals), synaptophysin 1 (1:1,000, Cat. 101 011, Synaptic Systems, Gottingen, Germany), Olig2 (1:250, Cat. 18953, IBL Co., LTD, Japan) and NG2 chondroitin sulfate proteoglycan (1:500, Cat. MAB5384, Chemicon International, Billerica, MA, USA).

### Electron microscopy

Cell cultures were fixed with 2% paraformaldehyde/2% glutaraldehyde in 0.13 M sodium cacodylate buffer, pH 7.4 at 37 °C and postfixed in 2% OsO_4_ for 2 h at 4 °C. Samples were dehydrated in a graded series of alcohol, infiltrated in propylene oxide/Spurr's and embedded in Spurr/s resin. Ultrathin sections were cut with a diamond knife, stained with uranyl acetate and lead citrate and examined by transmission electron microscopy.

### Electrophysiological recordings

Patch-clamp recordings of OPCs from monocultures or DRG-OPC co-cultures were performed in voltage-clamp mode 1–3 days after plating at room temperature and using an extracellular solution containing (in mM): 126 NaCl, 2.5 KCl, 1.25 NaH_2_PO_4_, 26 NaHCO_3_, 20 glucose, 5 pyruvate, 2 CaCl_2_ and 1 MgCl_2_ (95% O_2_ and 5% CO_2_). The intracellular solution contained (in mM): 120 K-gluconate, 5 NaCl, 3 MgCl_2_, 0.2 EGTA, 10 HEPES, 0.3 Na-GTP, 4 Na-ATP and 10 Na-phosphocreatine (pH≈7.3, 295 mOsm). To investigate the presence of fast inward AMPAR-mediated synaptic currents in OPCs of DRG-OPC co-cultures, DRG axons were stimulated while OPCs were recorded in voltage-clamp mode at a holding potential of −80 mV in co-cultures previously unstimulated and pre-stimulated electrically with or without BoNT/A. Holding potentials were corrected by a junction potential of −10 mV. Extracellular stimulation of DRG axons was performed using a bipolar electrode placed in a side compartment (0.5–5 V, 1–10 ms of duration). Single pulses or train of stimuli at a rate of 1, 10 and 100 Hz were applied for each neuron (Supplementary Fig. 2a).

Recordings were made without series resistance compensation. Series resistances were monitored during recordings and cells showing a change of >30% were discarded. Whole-cell recordings were obtained using Multiclamp 200B amplifier (Molecular Devices), filtered at 5 kHz and digitized at 10 kHz. Digitized data were analysed off-line using pClamp 10.1 software (Molecular Devices). Current densities for steady-state *I*–*V* relationships of OPCs were obtained by dividing the measured current amplitudes by cell capacitance. The amplitudes of inward sodium currents were measured at different voltage steps from +20 to −110 mV after leak subtraction using a MATLAB custom routine (MATLAB, MathWorks, Inc). The ohmic leak current for each potential was estimated by scaling the responses at most hyperpolarizing pulses. Means and data distributions of steady-state and inward sodium current densities, membrane resistance and capacitance were not different among treatments (*P*>0.05) (Supplementary Fig. 2d–g).

### Statistical analysis

Statistical significance was tested by analysis of variance in experimental designs involving multiple groups, followed by Dunnet's *post hoc* test for evaluating differences with respect to a control group and Fischer's comparison test for designs comparing differences among all groups. Two-sided Student's *t*-test was used for analysis of experimental designs with two groups, and a paired *t*-test was used in experiments in which repeated measures were made in the same sample. Data are displayed as means and s.e.m. For electrophysiological data, each data group was first subject to D'Agostino and Pearson normality test. According to the data structure (non-normally or normally distributed), the Kruskal–Wallis or two-way analysis of variance test was used for comparisons. Multiple Kolmogorov–Smirnov test was used to compare distributions of data. All statistical analysis and plotting were performed with Minitab version 12 (State College, PA, USA), SigmaPlot and GraphPad Prism 5.00 software (GraphPad Software Inc., USA).

## Additional information

**How to cite this article:** Wake, H. *et al.* Nonsynaptic junctions on myelinating glia promote preferential myelination of electrically active axons. *Nat. Commun.* 6:7844 doi: 10.1038/ncomms8844 (2015).

## Supplementary Material

Supplementary Information Supplementary Figures 1 and 2
