# Supplementary material for: Nonsynaptic junctions on myelinating glia promote preferential myelination of electrically active axons
Source: Nat Commun. 2015 Aug 4;6:7844. doi: 10.1038/ncomms8844 (PMC4532789; doi:10.1038/ncomms8844)
Supplement: Supplementary Information — Supplementary Figures 1 and 2 [file ncomms8844-s1.pdf]

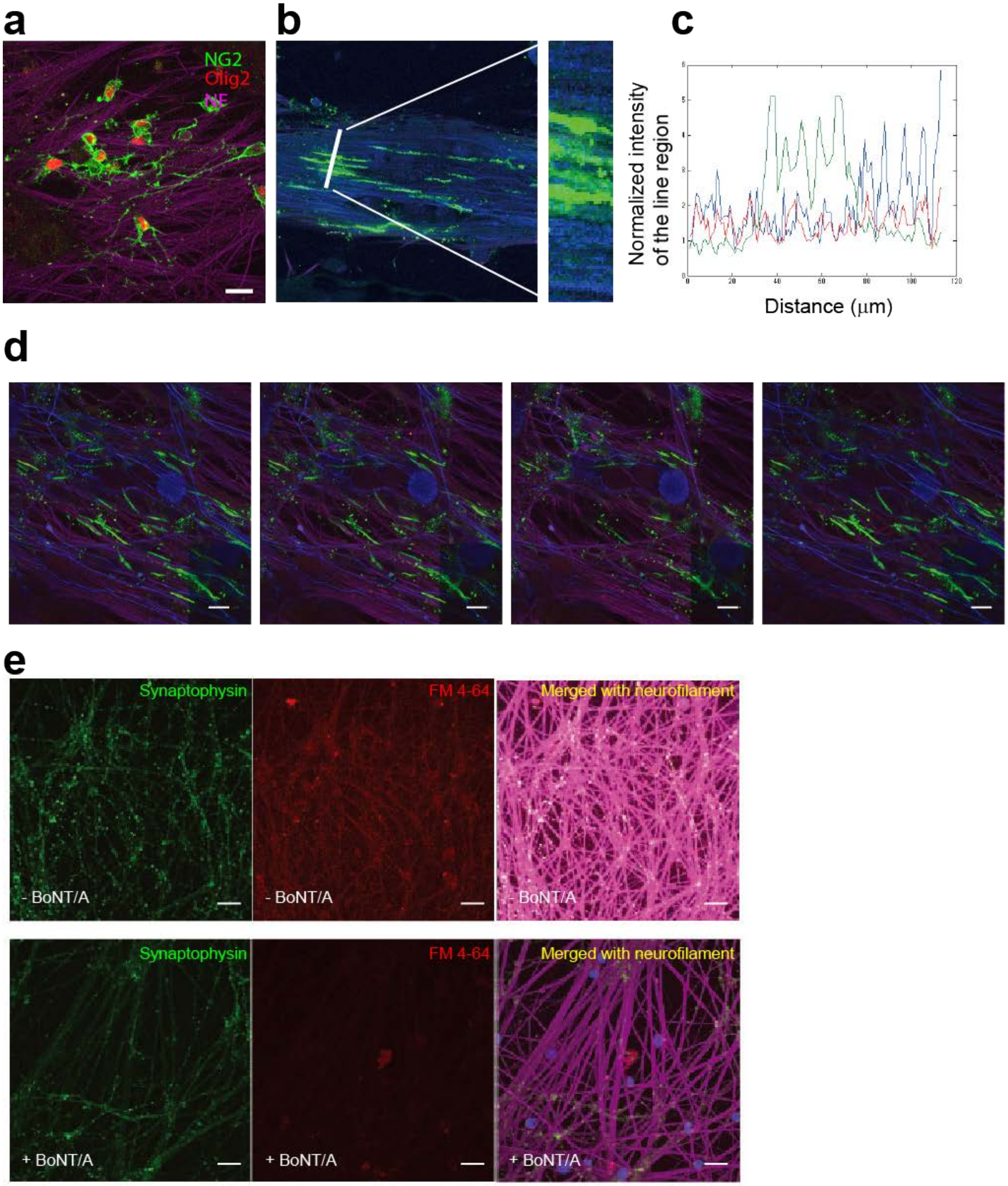
a
b
c
d
e
NG2
Olig2
NF
Normalized intensity of the line region
Distance (μm)
Synaptophysin
FM 4-64
Merged with neurofilament
- BoNT/A
+ BoNT/A

Supplementary Fig 1 Myelination occurs preferentially on electrically active axons releasing synaptic vesicles.

(**a**) OPCs were cocultured with DRG neurons for 24 hr and the cultures were fixed and stained by immunocytochemistry for NG2 (green) and Olig2 (red) and neurofilament (NF, purple). (**b**) Left panel: DRG axons in which exocytosis of synaptic vesicles was blocked by prior treatment with BoNT/A and stained with cell tracker (blue) were co-cultured with normal axons (unstained) and allowed three-weeks to form myelin (MBP, green). Right panel: High magnification. (**c**) Quantification of fluorescence intensity along the 120 μm line scan in **a** showing co-localization of myelin (MBP stain, green) with unstained axons. The intensity of green fluorescence (MBP stain of compact myelin, plotted in green) did not overlap with the intensity of blue fluorescence in axons treated with BoNT/A (blue line), indicating myelination preferentially occurred on non-colored axons (red line). **(d)** Z axis series of confocal images showed preferential myelination on axons releasing synaptic vesicles (non-blue-stained axons). Scale bar, 20 μm. **(e)** Vesicle recycling along DRG axons was reduced with BoNT/A treatment. Compare upper panel synaptophysin and FM 4-64 staining of vesicles with lower panel (green, synaptophysin; red, FM 4-64 dye). Scale bars 20 μm.

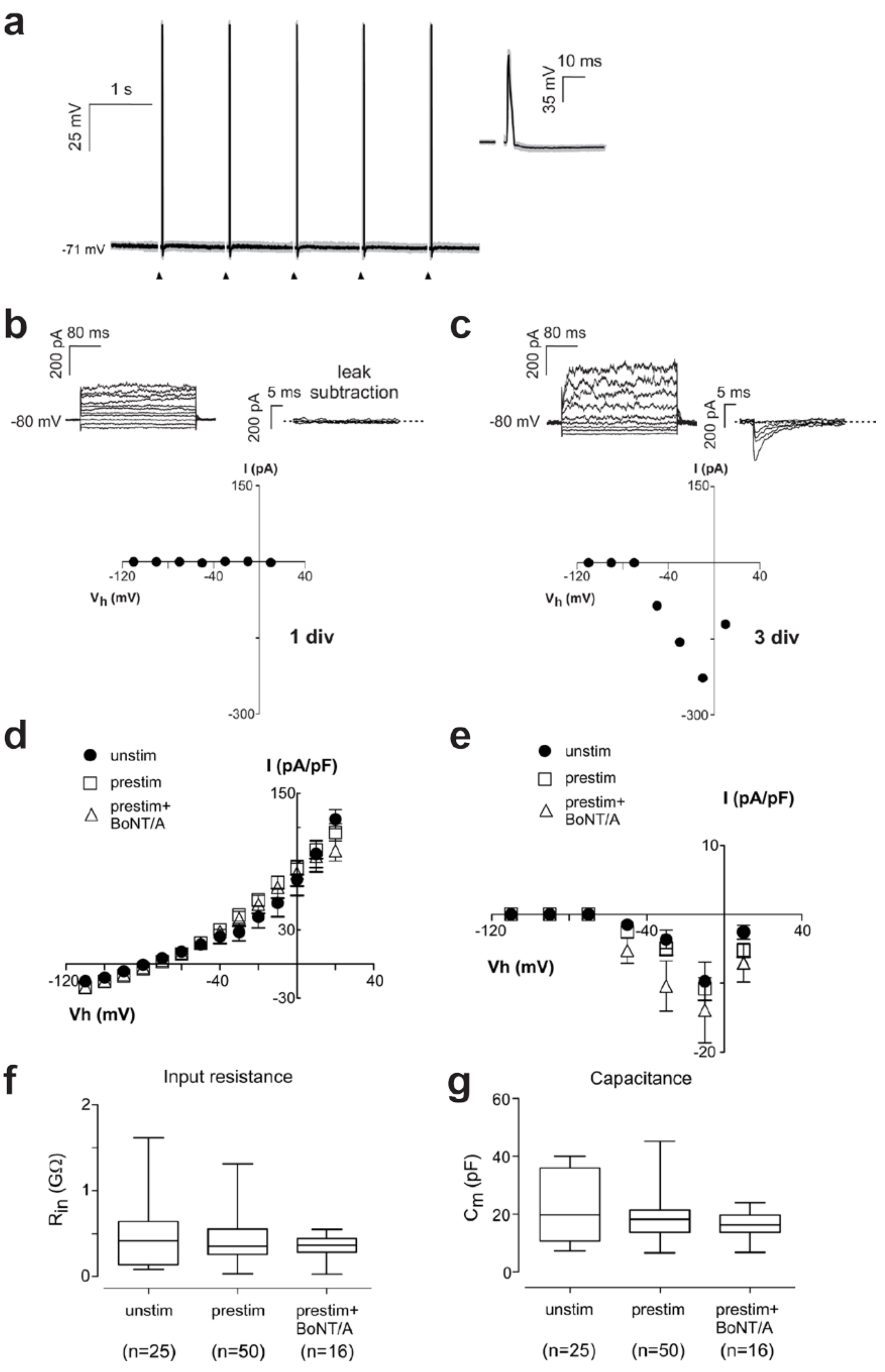

a
1 s
25 mV
10 ms
35 mV
-71 mV
b
80 ms
200 pA
-80 mV
5 ms
200 pA
leak
subtraction
I (pA)
150
-120
-40
40
Vh (mV)
1 div
-300
c
80 ms
200 pA
-80 mV
5 ms
200 pA
I (pA)
150
-120
-40
40
Vh (mV)
3 div
-300
d
unstim
prestim
prestim+
BoNT/A
I (pA/pF)
150
30
-120
-40
40
-30
Vh (mV)
e
unstim
prestim
prestim+
BoNT/A
I (pA/pF)
10
-120
-40
40
Vh (mV)
-20
f
Input resistance
Rin (GΩ)
2
1
0
unstim
prestim
prestim+
BoNT/A
(n=25)
(n=50)
(n=16)
g
Capacitance
Cm (pF)
60
40
20
0
unstim
prestim
prestim+
BoNT/A
(n=25)
(n=50)
(n=16)

Supplementary Figure 2. Action potentials elicited in cultured DRG neurons and OPC intrinsic properties.

(a). Extracellular train stimulation (1 Hz) of DRG axons triggers reproducible antidromic action potentials (APs) in a recorded neuron of a pre-stimulated co-culture treated with BoNT/A (13 sweeps in gray and average in black). Inset shows the first AP of the train. We confirmed that DRG neurons were able to elicit APs in all conditions (unstimulated neurons n=3 neurons from 2 dishes; prestimulated with or without BoNT/A n=4 neurons from 3 dishes). DRG neurons were recorded in current-clamp mode in one side of the three compartment chamber while extracellular stimulation of DRG axons was performed using a bipolar electrode on opposite sides of the barrier between the central and middle compartments (0.5-5 V, 1-10 ms pulse duration). Average latency for the evoked APs was 3.7 ± 0.1 ms. (**b, c)** Currents elicited by voltage steps from +20 mV to -110 mV in two OPCs held at -80 mV at days 1 (**b**) and 3 (**c**) after plating in monoculture (top traces). I-V curves of OPCs in **b** and **c** are shown (bottom). Note the appearance of a sodium current in **c**. Number of OPCs with sodium currents: 0/8 cells from 3 dishes at 0-1 days and 5/11 cells from 4 dishes at 2-3 days. (**d**, **e)** Steady-state (**d**) and inward (**e**) I–V curves showing, respectively, the characteristic outward rectification and the inward sodium currents of OPCs in three different conditions (Two way ANOVA, p=0.4074 and p=0.4938 for steady state and inward currents comparison respectively). Current density was obtained by dividing current amplitudes by cell capacitance. (**f**, **g).** Medians of OPC membrane resistance (R_{in}) and capacitance (C_m) were not different among groups (Kruskal-Wallis Test, p=0.1463 and p=0.4975, respectively). Interquartile range (box), median (line) and total range (whiskers) are indicated. Data distributions were not different (Multiple Kolmogorov-Smirnov Test, p= 0.4253

and p=0.2986 for R_{in} and C_m respectively). Groups: unstim = unstimulated co-cultures, n=25 cells from 7 dishes; prestim = prestimulated co-culture, n = 50 cells from 17 dishes; prestim+BoTN/A = prestimulated co-culture treated with BoTN/A, n=16 cells from 6 dishes.
